# Lymphangiectasis of lower limb: A rare challenging case

**DOI:** 10.4103/0970-0358.59292

**Published:** 2009

**Authors:** Visweswar Bhattacharya, Biswajit Mishra, Partha Sarathi Barooah, Gaurab Ranjan Chaudhuri, Siddhartha Bhattacharya

**Affiliations:** Department of Plastic Surgery, Institute of Medical Sciences, Banaras Hindu University, Varanasi - 221 005, U.P, India

**Keywords:** Lower limb, lymphangiectasis

## Abstract

Lymphangiectasis usually occurs in the viscera. Involvement of the lower limb is very rare. It is difficult to establish the diagnosis without detailed investigations. Clinical features are peculiar and may mimic lymphoedema of different origins which needs to be ruled out. Contrary to the expectation, the post-operative result is excellent in the long-term follow-up.

## INTRODUCTION

Lymphangiectasis means dilatation of lymphatic channels. It is commonly encountered in pulmonary, intestinal and retroperitoneal tissues. The involvement of a lower limb is very rare. Although few simulating cases have been reported, their clinical presentation as well as the surgical outcome were different from the present one. We have encountered a case that posed a difficult diagnostic puzzle. However, after thorough investigations, we established the diagnosis as Lymphangiectasis by excluding the other differential diagnoses, e.g. lymphoedema, lymphangioma and lymphangiomatoses. Staged excision of the lesion led to an excellent outcome without any recurrence after 3 years.

## CASE REPORT

**Figure 1:**
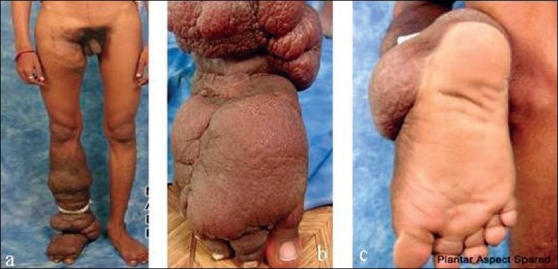
(a-c) Multiple lobulated masses mimicking Grade IV lymphoedema involving the groin, below knee and dorsum of foot, sparing the mid thigh and sole

A 20-year-old man presented with multiple lobulated growths of the right lower limb, lymphorrhea and disparity in limb girth. In the first 12 years of life, the limb was proportionate. Thereafter, for next 8 years, the right lower limb overgrew progressively which was painless. There were intermittent episodes of infection which subsided with antibiotic therapy and local care. The patient became physically and socially handicapped and lost his job. On inspection, the right lower limb below knee and dorsum of foot had multiple lobulated masses mimicking grade IV lymphoedema. The skin over the swelling was rugouse with dark pigmentation. The sole was spared. There was another lesion in the upper thigh extending to the groin. The lower two-third of the thigh was spared [Figure 1a–c].

**Figure 2:**
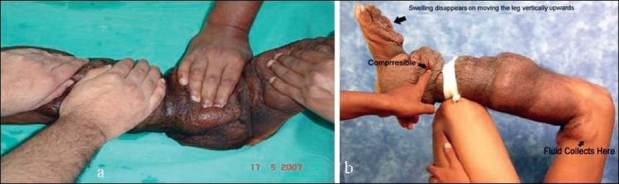
(a) Freely compressible lesion. (b) On limb elevation and compression, the fluidcollected at the lower thigh

On palpation, it was soft, non-tender, compressible and non-pulsatile [Figure 2a]. There was a significant reduction in size on elevating the limb. On compression, we could see and feel the fluid gushing towards a pouch in the lower thigh region. Again on lowering the limb, the fluid could be felt rushing back to below knee region – a peculiar feeling [Figure 2b]. Aspiration yielded an amber-colored fluid.

**Figure 3:**
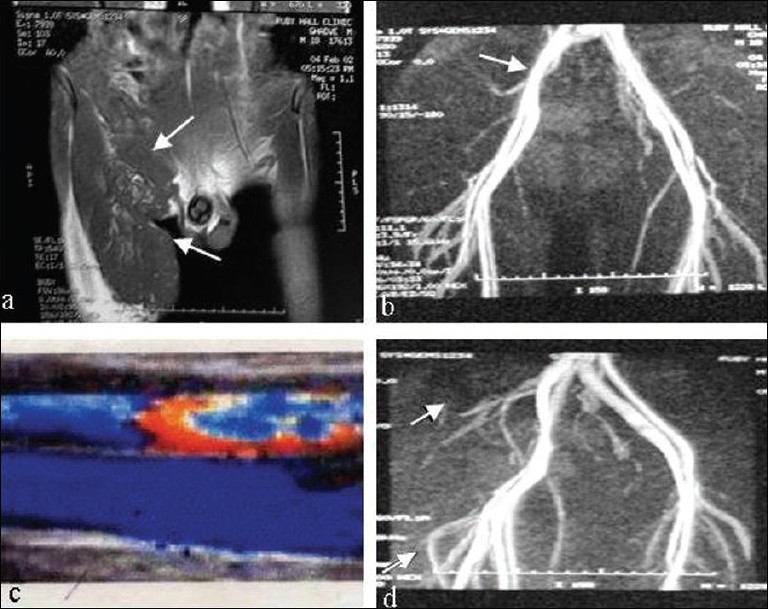
(a) MRI showing extensive mass in the subcutaneous tissue of right thigh with retroperitoneal extension. (b) MR angiogram showing narrowing of right iliac and femoral vessels due to extrinsic compression. (c) Colour Doppler showing normal caliber of vessels with optimum flow. (d) lymphangiography revealing dilatation of lymphatic channels in leg and inguinal region (Rt.).

The differential diagnoses in our mind were lymphoedema, lymphangioma, lymphangiomatosis and lymphangiectasis. The patient was subjected to detailed investigation. The MRI of lower limbs and pelvis showed an extensive mass involving predominantly the subcutaneous tissue of right thigh with retroperitoneal extension. There was encasement of femoral and iliac vessels [Figure 3a]. MR angiography showed narrowing of right iliac and femoral vessels due to extrinsic compression. There was no intrinsic vessel involvement [Figure 3b]. The colour Doppler study showed normal patency of the limb vessels with normal flow [Figure 3c]. Final diagnosis was established by lymphangiography and lymphoscintigraphy which revealed marked dilatation of lymphatic channels in leg and inguinal region [Figure 3d]. The cytology of the aspirated fluid showed mature lymphocytes in a protein-rich background. Thus, the diagnosis of lymphangiectasis was confirmed.

**Figure 4:**
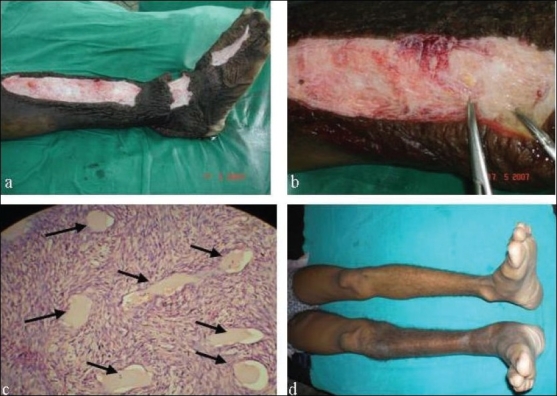
(a) Below knee incisions at three levels. (b) Dense dilated lymphatic network from dermis to deep fascia. (c) Histology showing lymphatic tissue with dilated lymphatic channels. (d) Three years follow-up with complete recovery.

A two-staged excision was planned for the leg and thigh separately. The below knee lesions were excised from the dorsum of foot, ankle and pretibial region by three individual incisions. Soon after incision, straw-coloured lymph started flowing freely. About 2 liters of fluid was completely squeezed out. There was hardly any delineation of subcutaneous tissue from dermis to deep fascia. Every structure was replaced by a dense, dilated network of lymphatic channels [Figure 4a,b]. It was excised in full depth along with a portion of excess skin. The wound was closed primarily, and pressure dressing was applied. Stitches were removed on 12^th^ day. After 6 months, the groin lesion was excised. No attempt was made to excise the retroperitoneal mass.

The histopathology of the excised specimen confirmed it to be lymphatic tissue with interconnected, dilated lymphatic spaces [Figure 4c]. On regular follow up, there was no recurrence after 3 years. The result was excellent although hyperpigmentation of the skin persisted [Figure 4d].

## DISCUSSION

Lymphangiectasis means dilatation of lymphatics with collection of excess lymph. It usually involves viscera e.g. lungs, intestine or retroperitoneum, the later manifest in the form of chyluria.[1–3] There had been reports of giant congenital cavernous lymphangioma of limb with bony lesions and extension into pelvis and retroperitoneum.[4] Lower limb lymphangiomatosis with soft tissue involvement alone has been described where the age of onset was 11 and 12 years in two cases.[5] In our case, the age of onset was 12 years. At 20 years he presented to us with elephantiasis of the right lower limb. A rare form of giant congenital lymphangiomatosis of the lower limb with ostolytic lesions in femur and tibia on roentgenographic skeletal survey has been reported but without any systemic involvement.[6] In this case, the limb bones were spared. During last 40 years, in our lymphoedema clinic, we have managed a large number of secondary lymphoedema of various grades involving the lower limb. A majority of them were of filarial origin. However, we never found such variation of clinical presentation. The free flow of large volume of lymph from one area to another has never been reported. All the components of clinical features were unique. Even vascular malformation could not be ruled out. Thus, it posed difficulty in diagnosis and management. Since clinical evaluation did not lead to the exact nature of the lesion, we subjected him to detailed investigations that proved to be very useful to ascertain the definitive diagnosis of lymphangiectasis. Since no definitive surgical procedure is described for such a lesion, we planned staged excision. On exploration, the extensive dense lymphatic network soft in consistency was a remarkable feature in contrast to the secondary lymphoedematous tissue that is very firm due to fibrosis following repeated infection. However, it could be easily excised along with excess skin. The primary suturing followed by pressure dressing led to primary wound healing. Encouraged by result of the first stage, we excised the upper thigh lesion after 6 months that also proved very satisfactory. We have followed the patient for more than 3 years now and the initial good result still persists without any evidence of recurrence.

Most cases of lymphangiectasis have extensive visceral involvement and poor prognosis. In this variant, it is limited almost exclusively to soft tissue of the lower limb and has good prognosis. This rare case posed unpredictable diagnosis with excellent surgical outcome.
